# Effects of Inclusion of Fresh Forage in the Diet for Lactating Buffaloes on Volatile Organic Compounds of Milk and Mozzarella Cheese

**DOI:** 10.3390/molecules25061332

**Published:** 2020-03-15

**Authors:** Raffaele Sacchi, Andrea Marrazzo, Felicia Masucci, Antonio Di Francia, Francesco Serrapica, Alessandro Genovese

**Affiliations:** Department of Agricultural Sciences, University of Naples Federico II, Via Università 100, 80055 Portici (NA), Italy; draven88@hotmail.it (A.M.); difranci@unina.it (A.D.F.); francesco.serrapica83@gmail.com (F.S.); alessandro.genovese@unina.it (A.G.)

**Keywords:** buffalo milk, buffalo Mozzarella, fresh forage, silage, volatile organic compounds, SPME-GC/MS

## Abstract

This paper aimed to assess the effects of feeding fresh forage on volatile organic compounds (VOCs) of buffalo milk and mozzarella cheese. Sixteen lactating buffaloes were equally allotted into two groups fed diets containing (experimental (Exp) group) or not (control (Ctl) group) 20 kg/d of fresh sorghum. Milk from the groups was separately collected and transformed in the traditional ‘Mozzarella di Bufala Campana’ Protected Denomination of Origin (PDO). Three batches of mozzarella were produced for each diet and they were analyzed, along with the two bulks of milk, for VOC composition, by using solid phase micro-extraction (SPME) coupled with gas-chromatography/mass spectrometry (GC/MS). The use of fresh forage increased the levels of long chain fatty acids along with the contents of aldehydes, and this could be responsible for an increase in green notes of milk. The use of the Ctl diet, containing a higher proportion of silage, increased the ketones, acids, and esters, which are compounds that could raise the cheese and fruity notes of milk. The mozzarella was less affected by the dietary treatment than milk. The use of fresh forage (sorghum) enhanced the green notes of milk and induced a few changes in the VOC profile of the typical PDO Mozzarella di Bufala Campana cheese, that were nonetheless detectable by sensory analysis. The low level found for butanoic acid, 2,3-pentanedione, and propyl acetate in mozzarella cheese obtained with fresh forage diet can lead to perceive less the olfactory notes of cheese, cream, and fruit.

## 1. Introduction

In Italy, milk from water buffaloes (*Bubalus bubalis*, Mediterranean type) is produced in specific areas of Lazio and Campania Regions. In recent decades, buffalo farming has been involved in an intensification of rearing and feeding techniques, so that total mixed rations (TMR) based on silages, hays, and concentrates are commonly used throughout the year, with limited or no use of fresh forage or pasture. This feeding system, made necessary also by the limited farm size, allows increasing both productions of milk/cow and the number of animals raised on farm, and ultimately the farm income. Buffalo milk is almost completely transformed in mozzarella, a fresh cheese covered by the EU Protected Denomination of Origin (PDO) “Mozzarella di Bufala Campana” (Commission Regulation EC No 103/2008) [1,2,3]. This cheese presents peculiar sensory properties and flavor, playing an important role for the product specificity and consumer acceptability, also in relationship to volatile organic compounds (VOCs) arising from raw buffalo milk and cheese-making technology. The odor of raw buffalo milk is indeed very different from other ruminants’ milk, due to specific VOC profiles, being 1-octen-3-one, nonanal, indole, 3-hydroxy-2-butanone, 3-methyl-2-buten-1-ol, 2-octanone, 2-hydroxy-3-pentanone, and heptanal, the main buffalo milk odor-active VOCs [4,5,6]. Native milk VOCs arise mainly from the animal diet, some of them already present in the feeds, others transformed during the digestion, absorbed, and directly transferred into milk via rumen and/or via the respiratory tract [7,8,9,10]. The mozzarella cheese flavor instead arises mainly from the interaction among native milk components, microbial starters, rennet, cheese making technology, and secondary microflora enzymes [5]. 

It is well known that the inclusion of fresh forage in ruminants’ diet may produce healthier milk, since it is richer in conjugated linoleic acid and n-3 fatty acids [11,12], and might also increase the casein and protein content, an issue of importance for mozzarella yield [13]. In this regard, the increased consumer interests about the nutritional and health properties of foods and their contribution to the human diet could create new market opportunities for mozzarella cheese [14]. The variations in milk composition induced by the cow feeding can be reflected in milk and cheese flavor, according to the metabolic pathways involved in aroma compound production [15]. Although a certain number of studies examined how the forage type can affect VOCs and organoleptic properties of cow milk and cheese [16,17,18,19,20,21,22], to the best of our knowledge, only one study has examined this topic for buffalo dairy products, finding that, compared to hay, the use of ensiled forage significantly increased the decanal and nonanal, alcohols, ketones, and terpenes of mozzarella cheese [23]. 

The hypothesis of this study is that the inclusion of fresh-cut forage in a TMR for lactating buffaloes under ordinary farming conditions may also influence, beside the fatty acid composition, the VOC profile of milk and cheese. We used sorghum, a forage species that is spreading in intensive Italian dairy farming, since, compared with maize silage, it is more flexible (it can be used both fresh and ensiled), and needs lower inputs of water and nitrogen fertilizer [24,25]. Therefore, following up on a previous study describing the effect of the use of fresh forage on mozzarella fatty acid composition and sensory properties [26], this paper aimed to examine the VOC composition of buffalo raw milk and mozzarella produced by including 20 kg of fresh sorghum in the diet for lactating buffaloes. 

## 2. Results

The experimental (Exp) and control (Ctl) diets differed in terms of presence of fresh sorghum and incidence of ensiled forages (19 vs. 28 kg/d as fed, respectively), but had similar compositions, in particular in regards to the protein and energy content (Table 1). 

In Table 2 are summarized selected data on milk composition and odor/flavor attributes of mozzarella cheese of the two dietary treatments. As discussed in Uzun et al. [26], no effect of diet was found for milk yield and chemical composition, as a result of the fact that the Exp and Ctl diets were kept isonitrogenous and isoenergetic. By contrast, compared to the Ctl, the fatty acid (FA) profile of the Exp mozzarella was characterized by increased levels of monounsaturated (MUFA) and polyunsaturated FA (PUFA). From a sensorial point of view, the Exp mozzarella had a lower overall odor, overall flavor (*p* < 0.05), milk flavor (*p* < 0.01), and whey odor/flavor (*p* < 0.05). The milk FA composition was not assessed, but it might be gathered from that of mozzarella, since it has been largely demonstrated that the FA of milk and fresh cheese do not significantly differ [27,28,29,30]. 

**Figure 1:**
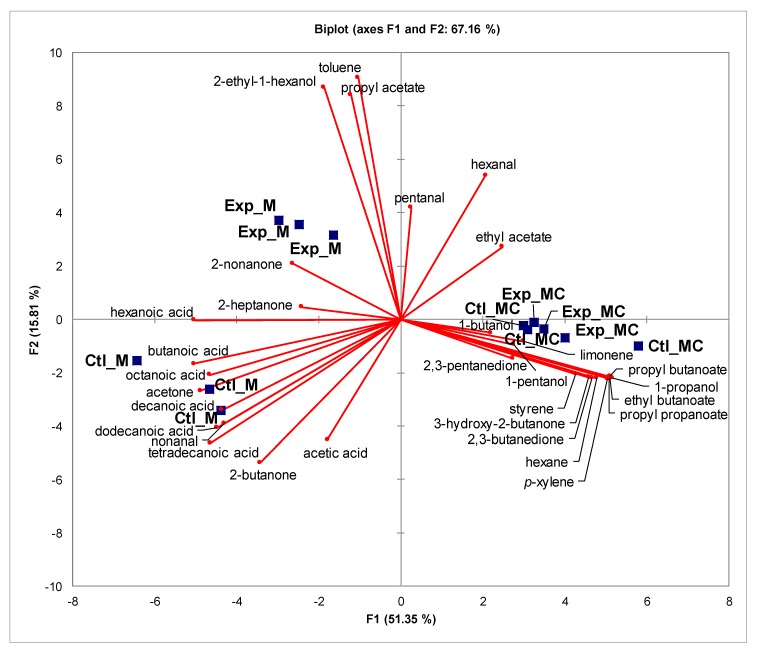
Principal component analysis (PCA) of volatile compounds identified in milk (M) and mozzarella cheese (MC) samples obtained from buffaloes fed total mixed ration with (Exp) or without (Ctl) fresh sorghum.

In Table 3, the VOCs identified in the milk and mozzarella cheese samples are given, and Table 4 lists their sensory descriptors and odor thresholds, based on the available literature [5,6,16,20,31,32,33,34,35,36,37,38,39,40,,41,42,43]. The performed analysis allowed us to identify in 18 different VOCs in the headspace of milk samples recognized as odor-conferring molecules. Except for hexanoic acid, 2-heptanone, and 2-nonanone, the dietary treatment influenced all milk VOCs in terms of quality and quantity. In detail, the presence of pentanal, hexanal, 2-ethyl-1-hexanol, ethyl acetate, propyl acetate, and toluene characterized the Exp milk, whereas acetic acid and 2-butanone were found only in Ctl milk. The most abundant volatile compounds were the free fatty acids (FFA) that were almost all statistically higher in Ctl milk, namely tetradecanoic acid (*p* < 0.001), dodecanoic acid (*p* < 0.01), decanoic acid, butanoic acid (*p* < 0.05), and octanoic acid (*p* < 0.1). In similarity, acetone and 2-butanone, among ketones, and nonanal, among aldehydes, were higher in Ctl milk. Overall, in terms of chemical families, acids and ketones were higher in Ctl milk, whereas the inclusion of fresh forage led to the formation of alcohols, esters, hydrocarbons, and aldehydes, compounds that, except nonanal, were found only in Exp milk. Twenty-three different VOCs were detected in mozzarella cheese. For both Ctl and Exp mozzarella cheeses, hydrocarbons and esters were the most represented VOCs followed by ketones, aldehydes, alcohols, and finally, the FFA (Table 3). From a qualitative point of view, butanoic acid and 2,3 pentanedione were detected only in Ctl mozzarella, whereas 1-butanol and 1-pentanol were only in the Exp mozzarella. The transformation of milk into mozzarella led to a reduction of the differences between the two kinds of cheeses, and only propyl butanoate was found to be statistically higher (*p* < 0.01) in the Ctl mozzarella. These results indicate that the Exp cheese could be characterized by less intense ‘fruity’, ‘cream’, and ‘cheese’ notes than the Ctl sample (Table 4). The general picture of these findings is shown in the biplot of the principal component analysis (PCA) made on VOCs from milk and mozzarella cheese (Figure 1). The two main principal components explain 67.16% of the variance, with the Principal Component 1 discriminating milk and cheese samples. It is possible to note that the mozzarella cheese was less affected by the dietary treatment than milk. The milk samples were characterized mainly by the volatile fatty acids, acetone, 2-butanone, 2-heptanone, 2-nonanone, and nonanal. On the contrary, mozzarella cheese samples were characterized by esters (except propyl acetate), hydrocarbons (except toluene), alcohols (except 2-ethyl-1-hexanol), 2,3-butanedione, 2,3-pentanedione, and 3-hydroxy-2-butanone. The Principal Component 2 discriminated the Exp milk samples from the Ctl ones. The use of fresh forage led to have milk with a higher level of 2-nonanone, 2-ethyl-1-hexanol, toluene, pentanal, hexanal, and propyl acetate. In contrast, the Ctl samples were characterized by volatile fatty acids, acetone, 2-butanone, and nonanal. 

## 3. Discussion

Although investigation on the VOC profile of dairy products is a field largely investigated, the studies available for buffalo dairy products are scanty and, in addition, they adopted different analytical techniques, thus making the data comparison difficult [40]. Moreover, the cheese making processes, including the starter cultures and ripening, can also strongly influence cheese VOC profile [22,35,37,44], therefore further limiting any direct comparison. As a matter of fact, the VOCs of buffalo milk [4,45] and mozzarella [6,23], were numerically higher than those that we found, and the application of different extraction methodologies is probably the origin of these discrepancies. Nevertheless, although distillation and solid phase micro-extraction (SPME) are two non-comparable extraction procedures, our technique was able to detect two compounds (i.e., nonanal and 3-hydroxy-2-butanone) among the eight volatile compounds reported in the literature for buffalo and bovine mozzarella cheese [46]. From a sensorial point of view, these two compounds can exert a significant role in the aroma of mozzarella cheese, being characterized by a very low odor threshold. To the best of our knowledge, this is the first paper reporting the VOCs of buffalo milk and mozzarella cheese determined by the headspace solid phase micro extraction (HS-SPME), technique that allows a negligible sample manipulation, so potentially avoiding solvent residues, contaminants, or artifacts [17]. Hydrocarbons (toluene), aldehydes (hexanal and pentanal), alcohols (2-ethyl-1-hexanol), and esters (ethyl acetate and propyl acetate), were the key compounds of the Exp milk, while ketones and FFA were the key compounds for the Ctl milk. Except for FFA, these observations are in accordance with the trends indicated by Villeneuve et al. [20] for cow milk from three diets based on fresh forage, silage, or hay. Toluene can originate from β-carotene degradation [41] that is present in larger amount in fresh forages compared with hays or silage [12,47]. Therefore, the buffalo cows on Exp diet likely took more β-carotene, and this may explain the higher levels of toluene in milk. Pentanal and hexanal (‘cut grass’ flavor note) could derive from the higher levels in milk of PUFA, from which aldehydes are formed by the lipoxygenase (LOX) pathway [5,20]. However, there is not a strong explanation for the higher nonanal in Ctl milk, a compound that can be produced from MUFA by the LOX pathway [5], but also by lipid auto-oxidation [48]. Esters can be produced in the mammary gland by esterification of FFA with alcohols [46]. The Ctl diet, containing a higher quantity of silage, showed a significantly greater FFA content with chain lengths from 3 (acetic acid) to 14 (tetradecanoic acid) carbons. This observation is consistent both with the significantly higher content in milk/cheese of the FA from 4 to 10 carbons [26] and the enhancement of milk lipolysis detected in cows fed silage [49]. In addition, fresh forage is well known to reduce the lipolytic processes and free fatty acid release [50]. The acetic acid, might potentially derive from silage, being a main product of carbohydrate fermentation during the ensiling process, but very limited information is available on the carry-over of acids from silage to milk and cheese [51,52]. Ketones are common VOCs of most dairy products, in particular of surface-mould ripened and blueveined cheeses [40]. Ketones can arise from β-oxidation of saturated fatty acid [17], so their higher content in Ctl milk is in agreement with the FA composition. Moreover, even the higher content in Ctl milk of acetone and 2-butanone, which are reputed to originate from animal feeds [53], is consistent with the fact that silage is a main source of ketone compounds [54,55]. Overall, the differences in milk VOC profiles appear related to both the higher silage incidence in the Ctl diet and the presence of fresh forage in the Exp diet. The effect of the latter, however, might have been diluted by the presence of ensiled forages also in the Exp diet.

The cheese-making process greatly influenced the VOC composition of mozzarella cheese both in quantitative and qualitative terms. The high temperatures (80 °C) reached by the curd during the stretching phase might have determined a loss of volatiles. Although there are no studies comparing the VOC profile of buffalo milk and mozzarella produced from the same milk, this observation is consistent with the fact that milk pasteurization may considerably reduce the native milk flavor [44,53]. The action of the microflora during curd ripening may have produced new compounds at the expense of others. The activity of milk enzymes and bacteria strongly contributes to the flavor development of mozzarella cheese [54]. Indeed, the enzymatic hydrolysis of triglycerides to fatty acids and glycerol, mono-, or diglycerides (lipolysis), is essential to flavor development in many cheese varieties [55]. Alcohols in cheese can rise from lactose and amino acid metabolism, but also from the reduction of methyl ketones [56]. Esters may originate from acids by replacing acid hydrogen with an alcoholic radical during curd fermentation [57], and this process seems to be particularly induced by high level of free fatty acids in the Ctl milk. Acetoin (3-hydroxy-2-butanone) is the main ketone in mozzarella according to Moio et al. [46]. This compound, characterized mainly by ‘buttery’ and ‘woody’ sensory notes, may be formed from the enzymatic condensation of two molecules of acetaldehyde, or from the reduction of diacetyl (2,3-butanedione) catalyzed by the diacetyl-reductase released in cheese by *Streptococcus lactis* and *Streptococcus cremoris*, the main lactic acid bacteria of buffalo milk [58]. 

Although the dietary treatment little affected the VOC profile, panelists detected differences in flavor/odor between Exp and Ctl mozzarella. As pointed out by Bosset and Gauch [59] and Curioni and Bosset [40], this apparent contradiction may be due to the fact that the cheese flavor would seem to depend on a balance of all compounds present, rather than the level of each specific component. We conclude that the use of fresh forage (sorghum) enhanced the ‘grassy’ notes of milk and induced few changes in the VOC profile of the typical PDO Mozzarella di Bufala Campana cheese, but nonetheless detectable by sensory analysis. 

## 4. Materials and Methods 

### 4.1. Experimental Design, Animals, Diets and Cheese Production 

The experiment took place at a buffalo dairy farm (Campania region, southern Italy) producing Mozzarella cheese. Thirty-two homogenous lactating buffaloes were assigned to either a control (Ctl) or an experimental (Exp) group, balanced for number (n =16), milk yield (13.3 ± 1.5 kg/d), days in milk (110 ± 13 d), and parity (3.0 ± 1.3). The 2 groups were housed into two adjacent free-stall barns equipped with separate mangers and water troughs. The buffaloes were milked twice daily (h 0500–0017) in the same milking parlor furnished of pipeline milk system for the transport of milk to refrigerate (4 °C) tanks. The Ctl group was fed the standard TMR used by the farmer that was modified for the Exp group in order to contain 20 kg (about 26.5% of the diet on a dry matter (DM) basis) of fresh sorghum (*Sorghum bicolor* (L.) Moench x *Sorghum sudanense* (*Piper*) Stapf.; commercial hybrid Nicol, Pioneer Hi-Bred International, Johnston, IA, USA). The chosen rate of fresh forage inclusion allows to little change the daily feeding routine, has a limited impact on workload, and is able to extend the period of fresh forage availability. Sorghum was at the early milk stage, i.e., growth stage 5–6 according to the scale of Vanderlip and Reeves [60]. In this stage, the panicles are forming and all leaves are still green. Fresh cut sorghum was mixed into the TMR with the other ingredients. The two diets were kept isonitrogenous and isocaloric (Table 1) and were offered once daily (08:00 h) in an amount to occur 10% of orts. After 10 days of adaptation to diets, for 3 days, the milk from Ctl and Exp groups was separately collected at the evening and morning milking (approximately 200 kg of milk/group) and immediately sampled (n. 6 200 mL samples/group). Bulk milk from the two groups was transported to the dairy in separate stainless steel tanks under refrigeration (4 °C), and used to produce mozzarella cheese, at the same time in separate vats, according to the traditional procedure previously described [26]. Briefly, raw buffalo milk was gently heated (37 °C) and added with natural whey starter culture from the previous day manufacture and calf liquid rennet (75:25 chymosin/pepsin ratio, 235 IMCU/mL Clerici Linea Rossa ab/r Cadorago, Como, Italy). At curd formation, the coagulum was reduced to small particles (2–3 cm) and held under whey until the pH fell to 4.85, a value allowing manual stretching of curd into hot water (90–95 °C). Thereafter, the stretched curd was mechanically formed into 50 g of mozzarella cheese small balls that were cooled in water and placed in brine (10% NaCl). Three 500-g samples were taken for each group at each day of mozzarella production, packed in 100-g plastic bags containing diluted brine solution, and transferred to the laboratory where they were kept at −20 °C until analysis. Further details about experimental design, sorghum crop management practices, animals, feeds, and diets are given by Uzun et al. [26,61].

### 4.2. Milk and Mozzarella Chemical Analysis

The samples were separately analyzed by group and by day of production. Milk fat, protein, and lactose were determined on the same day of collection by means of a Milkoscan 605 (Foss Electric A/S, Foss Hillerød, Denmark). Feed and diets were analyzed according to the procedures of the Association of Official Analytical Chemists (AOAC International 2002) to determine dry matter (DM; method 930.15), ash (method 942.05), crude protein (CP; method 976.05), and ether extract (EE; method 954.02) [62]. The neutral detergent fiber (NDF) inclusive of residual ash was determined according to Van Soest et al. [63]. To determine the fatty acid composition of mozzarella cheese, fat was extracted using the Schmidt–Bondzynski–Ratzlaff method. The gas-chromatographic analysis was carried out by means of a DANI Master gas chromatograph (Dani Instrument SPA, Cologno Monzese, Milan, Italy) equipped with a Quadrex Bonded Cyanopropyl silicone capillary column (length 60 m, internal diameter 0.25 mm, film thickness 0.25 μm). Fatty acids were identified using the Supelco 37 Component FAME MIX (Supelco Co., Bellofonte, PA, USA). Sensory analysis of mozzarella was performed by a quantitative descriptive sensory analysis by using a panel consisting of 10 judges (6 females and 4 males) engaged and selected according to the standard ISO 8586-1 (ISO, 2012). The panelists developed and agreed on a specific vocabulary for mozzarella consisting of 19 attributes, 6 of them related to odor/flavor. Further details are in Uzun et al.’s study [26].

### 4.3. Milk and Mozzarella Volatile Organic Compound Analysis

The milk and cheese samples were separately analyzed by group and by day of production. The extraction of VOCs was performed using Headspace-SPME according to the procedure described by Genovese et al. [64] for milk, and Lee et al. [65], for mozzarella cheese. Briefly, 22.5 g of milk was transferred in a 50-mL bottle, then 30 µL of 2-methyl-3-heptanone (99% purity, Sigma-Aldrich, St. Louis, MO, USA), as internal standard (408 mg L^−1^, in water solution), and 2.75 g of sodium phosphate (NaH_2_PO_4_) (Sigma-Aldrich) were added. For the mozzarella cheese analysis, the samples at −20°C were finely grated and 22.5 g was transferred in a 100-mL bottle and suspended with 25 mL of distilled water. Thereafter, 2.75 g sodium phosphate and 50 μL of 2-methyl-3-heptanone (99% purity; Sigma-Aldrich), as the internal standard (408 mg L^−1^, in water solution), were added. The bottle was magnetically stirred for 5 min at 55 °C to homogenize the sample and accelerate the equilibrium of headspace volatile compounds between the milk matrix and the headspace. The SPME fiber was inserted through the Teflon septum in the bottle and exposed to the sample headspace 60 min at 55 °C during stirring. The SPME device (Supelco Co., Bellefonte, PA, USA) was equipped with a 50/3-μm thickness divinylbenzene/carboxen/ polydimethylsiloxane (DVB/CAR/PDMS) fiber coated with a 2-cm length stationary phase. The same type of fiber, highly efficient for cheese aroma analysis [66], was used for milk and cheese samples. Volatile compounds were analyzed by GC coupled with a mass spectrometer using a GC/MS Hewlett-Packard 6890N (Agilent Technologies, Palo Alto, CA, USA) equipped with a J&W HP-5MS capillary column (30 m × 0.25 mm i.d. × 0.25 μm Film Thickness; J&W Scientific, Folsom, CA, USA). The temperature was set at 40 °C for 2 min and increased from 40 to 160 °C at the rate of 6 °C/min, and from 160 to 210 °C at 10 °C min^−1^. The injector was kept at 250 °C. Helium was used as a carrier gas (0.9 mL min^−1^). The volatile compound thermal desorption was carried out by exposing the SPME fiber in the injector in splitless mode for 10 min. The compound identification was performed by comparing retention times and mass spectra obtained by analyzing pure reference compounds in the same conditions. Moreover, the identification was confirmed by comparing the mass spectra with those of the NIST data base. All chemical standards were supplied by Sigma-Aldrich. In a few cases, the pure chemical standard was not available, and the compound identification was labeled as tentative. Mass spectra were recorded at 70 eV. The source temperature was 230 °C and the interface temperature was 250 °C. Before using it, the fiber was conditioned at 270 °C for 1 h for the analysis. A blank test was performed before each analysis to prevent the release of undesirable compounds. Semi-quantitative data of milk volatile compounds was obtained by normalizing the peak areas of each compound with respect to the area of the internal standard peak. Peak area data were processed by the software Chemstation (Agilent Technologies, Palo Alto, CA, USA). For the analysis of milk, the coefficients of variation (%) of the chemical classes of acids, aldehydes, alcohols, esters, hydrocarbons, and ketones were 10.4, 19.2, 15.3, 13.6, 19.1, and 11.1, respectively. For the analysis of mozzarella cheese, the coefficients of variation (%) of acids, aldehydes, alcohols, esters, hydrocarbons, and ketones were 11.6, 7.7, 5.3, 7.5, 8.9, and 6.1, respectively. The analyses of volatile compounds were performed in triplicate and were repeated on three different samplings (n = 9). 

### 4.4. Statistical Analysis of Data

Data elaboration was performed with SAS statistical package version 8.1 (SAS Institute, Cary, NC, USA). Since the data were not normally distributed according to the Anderson Darling test (*p* < 0.05), the VOCs peaks were log_10_-transformed. One-way analysis of variance (Mixed procedure) was used to test the effect of diet (Ctl and Exp) on VOCs in milk and mozzarella cheese. The batch of production was utilized as the error term to test the main effect of diet. PCA was also applied to explore the correlation of data and the effect of the diet.

## Figures and Tables

**Table 1:** Ingredients (kg as feed/day per head) and chemical composition of the total mixed ration fed to buffalo cows.

Item	Exp ^a^	Ctl ^b^
Ingredients		
Maize silage	15.0	18.0
Grass silage	4.0	10.0
Fresh sorghum	20.0	-
Meadow hay	2.5	4.5
Concentrate ^c^	7.0	6.5
Mineral and vitamin premix	0.25	0.25
Forage to concentrate ratio, %DM	67.8	68.8
Chemical composition		
Dry matter, kg	15.5	15.0
UFL, n/kg DM	0.86	0.84
Crude Protein, %DM	14.85	14.16
NDF, %DM	45.09	42.17

Exp, experimental. Ctl, control. DM, dry matter. UFL, Unité Fourragère Lait: 1 UFL = 7.11 MJ/kg of net energy for lactation. NDF, neutral detergent fiber. ^a^ Total mixed ration containing 20 kg of fresh sorghum. ^b^ Total mixed ration containing no fresh sorghum. ^c^ Based (as-fed basis) on soybean meal (4.7%), maize meal (34.9%), sunflower meal (13.0%), and barley meal (5.2%).

**Table 2:** Milk and mozzarella characteristics (least square means), as affected by fresh sorghum dietary inclusion (data from Uzun et al., 2018 [26]).

Item	Exp ^a^	Ctl ^b^	SEM	*p*-Value
Milk yield, kg/head per day	9.01	8.7	0.26	0.558
Milk chemical composition, g/kg				
Fat	94.1	90.6	3.33	0.24
Protein	52.7	50.7	1.12	0.202
Fat/Protein ratio	1.86	1.86	0.04	0.93
Lactose	47.1	47.1	0.71	0.975
Mozzarella chemical composition, g/kg				
Fat	287.0	276.2	0.36	0.103
Protein	201.3	206.9	0.67	0.587
Fat/Protein ratio	1.43	1.34	0.06	0.37
SFA	75.01	79.01	0.17	< 0.001
UFA	24.95	20.95	0.11	< 0.001
MUFA	22.41	18.9	0.17	< 0.001
PUFA	2.96	2.00	0.03	< 0.001
Mozzarella odor/flavor intensities ^c^				
Overall odor	74.80	81.45	1.74	0.022
Overall flavor	57.70	65.15	2.08	0.03
Milk	59.25	70.05	2.05	0.004
Butter	51.75	48.90	3.12	0.533
Whey	17.45	24.60	2.04	0.032
Yogurt	21.35	20.60	1.70	0.762

SFA, saturated fatty acids. UFA, unsaturated fatty acids. MUFA, monounsaturated fatty acids. PUFA, polyunsaturated fatty acids. SEM, standard error of means. ^a^ Total mixed ration containing 20 kg of fresh sorghum. ^b^ Total mixed ration containing no fresh sorghum. ^c^ Intensity rated on a 0 (attribute not perceived) to 100 mm (attribute perceived as very strong) unstructured line.

**Table 3:** Volatile organic compounds in milk (n = 9) and mozzarella cheese (n = 9) samples obtained from buffaloes fed total mixed ration with (Exp) or without (Ctl) fresh sorghum.

		Milk			Mozzarella Cheese		
R.T.	Identified Compounds	Exp ^a^	Ctl ^b^	*p*-Value	Exp ^a^	Ctl ^b^	*p*-Value
	**Acids**	**3.32 ± 0.08**	**3.62 ± 0.08**	**	**1.15 ± 0.11**	**1.28 ± 0.11**	**NS**
2.01	Acetic acid	ND	1.63 ± 0.86	/	ND	ND	
4.53	Butanoic acid	2.55 ± 0.09	2.87 ± 0.09	**	ND	0.30 ± 0.05	/
9.40	Hexanoic acid	3.04 ± 0.1	3.2 ± 0.1	NS	1.15 ± 0.12	1.22 ± 0.12	NS
14.31	Octanoic acid	2.67 ± 0.11	3.1 ± 0.11	*	ND	ND	
18.81	Decanoic acid	1.97 ± 0.13	2.54 ± 0.13	**	ND	ND	
22.85	Dodecanoic acid ^(t)^	1.35 ± 0.09	2.04 ± 0.09	***	ND	ND	
25.85	Tetradecanoic acid ^(t)^	1.48 ± 0.04	2.11 ± 0.04	****	ND	ND	
	**Aldehydes**	**1.87 ± 0.16**	**1.77 ± 0.16**	**NS**	**1.82 ± 0.08**	**1.79 ± 0.08**	**NS**
3.10	Pentanal	1.36 ± 0.59	ND	/	1.27 ± 0.1	1.26 ± 0.1	NS
4.95	Hexanal	1.80 ± 0.75	ND	/	1.62 ± 0.1	1.57 ± 0.1	NS
12.73	Nonanal	1.46 ± 0.08	1.77 ± 0.08	**	1.10 ± 0.08	1.24 ± 0.08	NS
	**Alcohols**	**1.61 ± 0.24**	**ND**	**/**	**1.76 ± 0.04**	**1.62 ± 0.04**	*
10.72	2-Ethyl-1-hexanol ^(t)^	1.61 ± 0.24	ND	/	ND	ND	
1.91	1-Propanol ^(t)^	ND	ND		1.56 ± 0.04	1.62 ± 0.04	NS
2.70	1-Butanol	ND	ND		0.85 ± 0.34	ND	/
4.32	1-Pentanol	ND	ND		1.16 ± 0.14	ND	/
	**Esters**	**2.28 ± 0.51**	**ND**	**/**	**2.14 ± 0.05**	**2.24 ± 0.05**	**NS**
2.26	Ethyl acetate	1.38 ± 0.61	ND	/	1.37 ± 0.09	1.33 ± 0.09	NS
5.00	Ethyl butanoate	ND	ND		1.42 ± 0.06	1.48 ± 0.06	NS
3.37	Propyl acetate ^(t)^	2.23 ± 0.7	ND	/	1.63 ± 0.06	1.77 ± 0.06	NS
5.20	Propyl propanoate ^(t)^	ND	ND		1.18 ± 0.05	1.31 ± 0.05	NS
7.36	Propyl butanoate ^(t)^	ND	ND		1.48 ± 0.02	1.60 ± 0.02	***
	**Hydrocarbons**	**2.25 ± 0.75**	**ND**	**/**	**2.17 ± 0.08**	**2.19 ± 0.08**	**NS**
2.12	Hexane	ND	ND		1.79±0.14	1.76 ± 0.14	NS
4.25	Toluene	2.25±0.75	ND	/	1.61 ± 0.05	1.60 ± 0.05	NS
6.57	*p*-Xylene ^(t)^	ND	ND		1.06 ± 0.08	1.08 ± 0.08	NS
7.11	Styrene ^(t)^	ND	ND		1.28 ± 0.15	1.38 ± 0.15	NS
10.76	Limonene	ND	ND		1.21 ± 0.08	1.18 ± 0.08	NS
	**Ketones**	**2.59 ± 0.07**	**3.12 ± 0.07**	***	**2.11 ± 0.15**	**2.12 ± 0.15**	**NS**
1.69	Acetone	2.50 ± 0.06	2.92 ± 0.06	***	ND	ND	
2.13	2-Butanone	ND	2.59 ± 1.44	/	ND	ND	
7.16	2-Heptanone	1.70 ± 0.15	1.94 ± 0.15	NS	1.44 ± 0.13	1.49 ± 0.13	NS
12.43	2-Nonanone	1.85 ± 0.15	1.34 ± 0.15	NS	0.80 ± 0.15	0.85 ± 0.15	NS
2.06	2,3-Butanedione	ND	ND		1.53 ± 0.14	1.56 ± 0.14	NS
3.07	2,3-Pentanedione	ND	ND		ND	0.94 ± 0.4	/
3.27	3-Hydroxy-2-butanone	ND	ND		1.80 ± 0.16	1.71 ± 0.16	NS

Levels of volatile organic compounds evaluated using a solid-phase microextraction technique and expressed as log_10_ of the normalized level. See Materials and Methods for the description. R.T., retention time. ^a^ Total mixed ration containing 20 kg of fresh sorghum. ^b^ Total mixed ration containing no fresh sorghum. ^(t)^ Volatile compounds were tentatively identified. ND, not determined. NS, not significant (*p* > 0.1). Asterisks indicate significant differences between treatment-related samples (* *p* < 0.1; ** p < 0.05; *** *p* < 0.01; **** *p* < 0.001).

**Table 4:** Odor description and odor threshold in water (μg kg^−1^) of the identified volatile organic compounds in the assessed milk and mozzarella cheese.

Identified Compounds	Odor Threshold ^a^	Odor Description ^a^
**Acids**		
Acetic acid	0.022 [20]	Vinegar [6,16,20,31,32,33], vinegar sour [34], sharp [34], pungent [6]
Butanoic acid	0.001 [20]–240 [35]	Vomit [31,36], cheese [31,34,36,37], rotten [34], sharp [34], rancid cheesy [6], putrid [6], sweaty [6,36], buttery [37]
Hexanoic acid	3000 [35]	Sharp [34], goaty [34,36], pungent [6], blue cheese [6], sour [6]
Octanoic acid	3000 [35]	Goaty [6,37], waxy [6,37], soapy [6], musty [6], rancid [6,37], fruity [6], unpleasant [37], fatty [37], body odor [36,37]
Decanoic acid	10000 [35]	Waxy-sweet [34], rancid fatty [6]
Dodecanoic acid	10000 [35]	
Tetradecanoic acid	10000 [35]	
**Aldehydes**		
Pentanal	12–42 [38]	Woody [36], bitter oily [36]
Hexanal	4.5–5.00 [35]	Green apple [36], grassy [36]
Nonanal	1 [35]	Green [5,38,39], animals [5], grass-like [5,38,39], fatty [5,39,40], animal [39,40]
**Alcohols**		
2-Ethyl-1-hexanol		Spicy [5]
1-Propanol	9000 [37]	Pungent [6]
1-Butanol	500 [41]	Medicinal [40], floral [36], fragrant [36], fruity [36], sweet [36],
1-Pentanol	4000 [38]	Alcoholic [5], iodoform-like [5]
**Esters**		
Ethyl acetate	5000 [35]	Sticky [36], sweet [36]
Ethyl butanoate	1 [35]	Sweet [36], fruity [36]
Propyl acetate	57 [36]	
Propyl butanoate	18–124 [36]	Pineapple [35], sharp [35]
Propyl propanoate		Sweet [37], fruity [37], pineapple [37], apple [37], banana [37], bilberry [37], cider [37], cranberry [37], durian [37]
**Hydrocarbons**		
Hexane		
Toluene		Chemical [5,38], solvent [5,38], plastic [31]
*p*-Xylene		
Styrene	730 [36]	Plastic [5,31,38,41], rubber [31]
Limonene	0.0002 [20],10 [36]	Fruity [5,31,38], lemon [5,31,38], orange [20], citrus [36]
**Ketones**		
Acetone		
2-Butanone	500000 [35]	Varnish [5]
2-Heptanone	140–3000 [35]	Animals [5], blue cheese [5,38,40,41,42], mouldy [38], spicy [6,40,43], cinnamon [40,43]
2-Nonanone	5–200 [35]	Hot milk [5], smoked cheese [5], ketonic [5], varnish [5,38], fruity [31,40,42], floral [34,40,42]
2,3-Butanedione	0.000005 [20]	Butter [31,34,36], sweety [34], pastry [20]
2,3-Pentanedione		Buttery [6], cheesy [6], sweet [6], nutty [6], fruity [6], creamy [6], caramel [6]
3-Hydroxy-2-Butanone	0.0008 [20]	Woody [5,38], mildew [5,38], warm [5,38], buttery [31,36,40,42,43], creamy [43], weak earthy [43]

^a^ Sensory descriptors and the odor threshold in water reported for volatile compounds are from literature.
